# Evaluation of Hospital-Acquired Anemia and Contributing Factors in ICU Patients: A Retrospective Cohort Study

**DOI:** 10.3390/jcm15187065

**Published:** 2026-09-11

**Authors:** Alper Koç, Nesibe Aydoğdu, Elif Suyanı

**Affiliations:** 1 Division of Hematology, Department of Internal Medicine, Duzce University, Duzce 81620, Türkiye; 2 Department of Internal Medicine, Elazığ Fethi Sekin City Hospital, Elazığ 23270, Türkiye; nesibeaydogdu@gmail.com; 3 Department of Hematology, Adana City Training and Research Hospital, Adana 01230, Türkiye; elifsuyani@hotmail.com

**Keywords:** hospital-acquired anemia, intensive care unit, diagnostic phlebotomy, inflammation, retrospective cohort

## Abstract

**Objectives:** Hospital-acquired anemia (HAA) is an important clinical problem that can adversely affect outcomes, particularly in critically ill patients. This study aimed to determine the incidence of hospital-acquired anemia in adult ICU patients and to evaluate its relation with diagnostic phlebotomy procedures and other etiologic factors. **Methods:** Data from 1170 adult ICU admissions between 1 January and 31 December 2024 were retrospectively reviewed and 321 patients were included in the analysis. The median age of the study cohort was 72 years (range: 18–102), with 45.2% females (*n* = 145) and 54.8% males (*n* = 176). The median ICU length of stay was 6 days (range: 3–29). **Results:** HAA developed in 35.8% of patients, while a decrease in hemoglobin levels was observed in 80.4%. Patients who developed HAA had significantly longer ICU stays and higher ferritin, CRP, and diagnostic phlebotomy (DP) volumes, while admission hemoglobin levels were lower compared with those without HAA (all *p* < 0.05). In multivariate analysis, a longer ICU stay, higher ferritin levels, lower admission hemoglobin levels and greater DP volumes were independently associated with the development of HAA. **Conclusions:** HAA was common in critically ill patients and was associated with several clinical and laboratory factors, including inflammatory burden, baseline hemoglobin level, ICU length of stay, and diagnostic phlebotomy volume. These findings suggest that HAA is a multifactorial process, with ICU length of stay, inflammatory status, baseline hemoglobin levels, and diagnostic phlebotomy volume all potentially contributing to its development.

## 1. Introduction

Hospital-acquired anemia (HAA) is defined as anemia that develops during hospitalization in patients who are admitted with normal hemoglobin (Hb) levels. This condition was referred to as “nosocomial anemia” in earlier studies, with descriptive reports emerging in the 1970s [1]. In large cohort studies including unselected hospitalized populations, the incidence of HAA has varied from 25% to 75% depending on which Hb values were taken, regardless of discharge Hb values or the lowest Hb level measured during hospitalization [2]. Although the incidence of HAA appears to be around 20% in patients staying in general hospital wards, it affects almost all patients requiring intensive care [3,4]. Furthermore, anemia has been shown to be exacerbated in nearly 50% of patients who were already anemic at admission [5]. 

Although diagnostic phlebotomy (DP) is frequently emphasized as the major contributor to HAA in the literature, it does not fully account for the condition [6]. In addition, suppression of erythropoiesis and shortened red blood cell lifespan are important underlying mechanisms in the pathogenesis of HAA development [7]. Furthermore, older age, reduced bone marrow reserve, intensive care unit (ICU) stay, frequent DP, multiple comorbidities, and prolonged hospitalization have been identified as major risk factors [3].

In ICU patients, HAA occurs at much higher rates compared to other hospitalized populations [8]. In this study, we aimed to determine the frequency of HAA in ICU patients and its association with DP and other etiologic factors and to contextualize our institutional data within the current literature to better define the magnitude of the problem and underlying contributors.

## 2. Materials and Methods

### 2.1. Study Design and Patient Selection

This was a retrospective, single-center, observational cohort study. We reviewed the data of 1170 patients who were hospitalized in the Neurology and the Respiratory ICUs of Elazığ Fethi Sekin City Hospital between 1 January 2024, and 31 December 2024. The study population was identified by selecting patients with both admission Hb and discharge Hb measurements within the same hospitalization episode. Anemia was defined based on the World Health Organization (WHO) criteria: Hb < 12 g/dL in women and Hb < 13 g/dL in men. 

Patients who were anemic at the time of admission were excluded from the analysis. The other exclusion criteria were as follows: ICU stay shorter than 3 days or longer than 30 days; admission GFR < 30 mL/min/1.73 m^2^, as advanced renal dysfunction may independently affect hemoglobin levels and erythropoiesis; clinically documented active bleeding during hospitalization or follow-up (e.g., gastrointestinal, hemoptysis, and intracranial); missing data regarding admission Hb, discharge Hb, total DP volume, length of stay, or sex; and multiple ICU admissions within the study period (only one episode per patient was analyzed). The patients were eligible for inclusion if they met all of the following criteria: age ≥ 18 years; availability of both admission Hb and discharge Hb measurements within the same hospitalization episode; documented total diagnostic DP volume (mL); and an ICU stay duration of 3–30 days.

### 2.2. Data Collection

The following laboratory biomarkers were collected: Hb (g/dL), Hct (%), WBC (×10^3^/µL), and PLT (×10^3^/µL); liver function tests including ALT (U/L), AST (U/L), GGT (U/L), ALP (U/L), total bilirubin (mg/dL), and albumin (g/dL); renal function tests including serum creatinine (mg/dL) and eGFR (mL/min/1.73 m^2^); inflammation and tissue injury markers including C-reactive protein (CRP) (mg/L) and LDH (U/L); and nutritional markers including serum vitamin B12 (pg/mL), folic acid (ng/mL), serum iron (µg/dL), total iron-binding capacity (µg/dL), and ferritin (ng/mL). Hemogram parameters were recorded at admission (within the first 24 h) and immediately before ICU discharge, thereby allowing separate evaluation of admission and discharge measurements for each patient.

### 2.3. Diagnostic Phlebotomy Assessment

Furthermore, the types and counts of the sample tubes used for DP were recorded, including blood gas, biochemistry/serum, citrate (coagulation), EDTA (hemogram), and blood culture tubes. For each patient, the total DP volume (mL) was calculated by multiplying the number of tubes drawn by their institutional nominal volumes. The nominal volumes applied were (1) 1.0 mL for blood gas tubes, (2) 3.0 mL for biochemistry (serum) tubes, (3) 2.7 mL for citrate (coagulation) tubes, (4) 3.0 mL for EDTA tubes, and (5) 5.0 mL for blood culture tubes. 

Blood sampling was not performed according to a predefined or standardized protocol. The frequency and timing of blood collection were determined according to the patients’ clinical condition, laboratory monitoring requirements, and the attending physician’s clinical judgment.

The total DP volume was calculated using the following formula:

Total Phlebotomy Volume (mL) = 1.0 × (number of blood gas tubes) + 3.0 × (number of biochemistry tubes) + 2.7 × (number of citrate tubes) + 3.0 × (number of EDTA tubes) + 5.0 × (number of culture tubes).

### 2.4. Statistical Analysis

Normality of continuous variables was assessed using the Shapiro–Wilk test. Non-normally distributed variables were summarized as the median (minimum–maximum), and between-group comparisons were performed using the Mann–Whitney U test. Categorical variables were expressed as counts and percentages and compared using the Chi-square test or Fisher’s exact test where appropriate. Associations between clinical/laboratory variables and HAA development were examined using univariate and multivariate logistic regression analyses. Associations between clinical/laboratory variables and Hb decline were also assessed using univariate and multivariate logistic regression analyses. Additionally, ΔHb was calculated as the difference between admission Hb and discharge Hb (ΔHb = admission Hb − discharge Hb) and analyzed as a continuous variable using linear regression analysis after log transformation. The effects of age, sex, CRP, ferritin, and total diagnostic phlebotomy (DP) volume on ΔHb were evaluated.

All statistical tests were two-tailed, and a *p* < 0.05 was considered statistically significant. Analyses were performed using SPSS v24.0 (IBM Corp., Armonk, NY, USA).

Artificial Intelligence (AI)-Assisted Technology: ChatGPT (OpenAI, GPT-5.5.) was used solely for language editing, grammar correction, and improvement of readability during manuscript preparation and revision. It was not used for data analysis, statistical analysis, interpretation of results, or generation of scientific conclusions. All content was reviewed and verified by the authors.

## 3. Results

### 3.1. Patient Characteristics

Data from 1170 patients were reviewed and 321 patients met the eligibility criteria and were included in the final analysis. Baseline demographic and clinical characteristics of the study cohort are presented in Table 1. The median age of the study cohort was 72 years (range: 18–102), with 45.2% females (*n* = 145) and 54.8% males (*n* = 176). The median ICU length of stay was 6 days (range: 3–29).

At admission, the median eGFR was 82 mL/min/1.73 m^2^ (range: 30–217). The median WBC count was 8.46 × 10^3^/µL (range: 1.81–23.2), and the median PLT count was 228 × 10^3^/µL (range: 35–689). The median ferritin and CRP levels were 88 ng/mL (range: 4–1500) and 29 mg/L (range: 0.47–450), respectively. The median serum vitamin B12 level was 231 pg/mL (range: 50–1586). Of all patients, 84.4% had normal and 10.9% had low vitamin B12 levels. The median folate level was 7.79 ng/mL (range: 1.98–25); a total of 94.7% of patients had normal levels and 1.9% had low folate levels, and folate measurements were not available in 3.4% of patients.

Red blood cell transfusion was required in only three patients (0.9%). The median admission Hb was 14 g/dL (range: 12–19.2), while the median discharge Hb was 13 g/dL (range: 8–17.6). The median total DP volume was 58.2 mL (range: 17.4–554.8).

### 3.2. Incidence of Hemoglobin Decline and Hospital-Acquired Anemia

At ICU admission, the median Hb level was 14 g/dL (range: 12–19.2), which decreased to 13 g/dL (range: 8–17.6) at discharge. A decline in Hb was observed in 258 patients (80.4%), while 63 patients (19.6%) demonstrated stable or increased Hb levels. Based on WHO diagnostic thresholds, HAA was present in 35.8% of patients at ICU discharge. 

### 3.3. Comparison of Patients with and Without Hospital-Acquired Anemia

Patients were stratified according to the development of anemia during their ICU stay (Table 2). Hospital-acquired anemia developed in 115 (35.8%) patients. There were no significant differences between the groups with respect to age, sex, admission eGFR, and folate or vitamin B12 levels (all *p* > 0.05). However, patients with HAA were found to have significantly higher ferritin and CRP levels, a longer ICU length of stay and higher DP volumes. Also admission Hb level was lower in patients developing anemia (*p* < 0.05) (Table 2).

### 3.4. Logistic Regression Analysis of Factors Associated with Hospital-Acquired Anemia

In the univariate logistic regression analysis, ICU length of stay, ferritin, CRP, admission Hb levels and DP volume were found to affect the development of HAA (Table 3). And this affect was independent for ICU length of stay, ferritin level, admission Hb level and DP volume in multivariate analysis (Table 4) Age and sex were also included in the multivariable logistic regression model; neither variable was significantly associated with HAA development.

### 3.5. Comparison of Patients with and Without Hemoglobin Decline

Patients were stratified according to the presence of Hb decline during their ICU stay (Table 5). There were no significant differences between the groups with respect to age, sex, ICU length of stay, admission eGFR, admission Hb, or vitamin B12 levels (all *p* > 0.05). However, patients with Hb decline demonstrated significantly higher ferritin levels (98.5 vs. 60.5 ng/mL; *p* = 0.008) and CRP levels (40 vs. 18 mg/L; *p* = 0.01) compared with those without Hb decline. Conversely, folate levels were significantly higher in patients without Hb decline (9.25 vs. 7.25 ng/mL; *p* = 0.001). Total DP volume tended to be higher in the Hb-decline group compared with those without Hb decline (59.2 vs. 56.1 mL), although this difference did not reach statistical significance (*p* = 0.06). Other hematological parameters did not significantly differ between the groups.

### 3.6. Logistic Regression Analysis of Factors Associated with Hemoglobin Decline

In the univariate logistic regression analysis, ferritin levels were not associated with Hb decline (OR = 1.001; 95% CI: 0.999–1.002; *p* = 0.387). In contrast, higher CRP levels were significantly associated with increased odds of Hb decline (OR = 1.006; 95% CI: 1.001–1.010; *p* = 0.008) (Table 6). 

Total DP volume also showed a significant univariate association with Hb decline (OR = 1.011; 95% CI: 1.001–1.022; *p* = 0.027). In the multivariate analysis including CRP and DP volume, CRP remained an independent predictor of Hb decline (OR = 1.005; 95% CI: 1.001–1.009; *p* = 0.023) (Table 7), whereas DP volume did not retain statistical significance (OR= 1.009; 95% CI: 0.999–1.020; *p* = 0.068).

To further evaluate the extent of Hb reduction during ICU stay, the change in Hb levels between admission and discharge was additionally analyzed as a continuous variable. ΔHb was analyzed as a continuous variable using linear regression analysis after log transformation. The effects of age, sex, CRP, ferritin, and total diagnostic phlebotomy (DP) volume on ΔHb were evaluated. Total DP volume was significantly associated with ΔHb (*p* < 0.001; 95% CI: 0.001–0.0001).

## 4. Discussion

HAA is associated with adverse outcomes such as reduced oxygen-carrying capacity, increased cardiopulmonary burden, prolonged ICU and hospital length of stay, increased healthcare costs, and higher post-discharge mortality and morbidity. In the study by Koch et al., which included 188,447 patients, HAA developed in 74% of cases, and moderate-to-severe anemia was reported to be significantly associated with increased mortality, length of stay, and costs [9]. Similarly, in a multicenter study evaluating 11,309 patients, severe HAA was found to be associated with marked increases in 30-day mortality and readmission rates [2]. These findings highlight the clinical importance of identifying modifiable causes, especially in ICU patients. From this point of view we also studied the development of anemia in ICU patients and found that HAA was detected at a rate of 35.8% at discharge, while 80.4% of the patients experienced Hb decline during their ICU stay. This rate of HAA is lower than the previously reported prevalences of approximately 60–70% in critically ill patients [10]. This discrepancy could be explained by the strict exclusion criteria such as excluding patients with anemia, significant renal dysfunction or clinically evident bleeding, all of which resulted in a more hematologically stable population.

Diagnostic blood sampling is an important and modifiable cause of HAA development [11]. Due to frequent laboratory testing and the use of multiple sample tubes in the ICU, daily iatrogenic blood loss can reach 40–70 mL [2,12]. Various studies have demonstrated that this DP-related loss accelerates Hb decline and increases the need for transfusion [13,14]. We also evaluated the impact of DP on HAA and Hb decline. İn line with the literature, DP volume was shown to be significantly higher in patients developing HAA; furthermore, it was shown to be independently associated with HAA after adjustment for ICU length of stay and ferritin, CRP, and admission Hb levels in this cohort. On the other hand although patients who experienced a decrease in Hb level had higher phlebotomy volumes (59.2 vs. 56.1 mL), this difference did not reach statistical significance (*p* = 0.06). The weaker-than-expected association between phlebotomy volume and Hb decline may be explained by unmeasured blood losses that routinely occur in ICU settings. It has been frequently emphasized in the literature that the true magnitude of phlebotomy-related blood loss likely exceeds the recorded volume due to common practices such as discarding blood during arterial and venous catheter flushing, repeated sampling for coagulation and blood gas analyses, drawing larger blood volumes than necessary, and discarding unused samples [14,15]. Another point that needs to be addressed is quite high initial Hb levels in the cohort. The unknown etiology of the high initial Hb levels might prevent the precise evaluation of the relation between Hb decline and DP volume. For instance a high Hb level due to dehydration would drop promptly after hydration and this decline would be attributed to a small amount of DP.

The fact that anemia develops within the first 72 h in most critically ill patients and that transfusion requirements frequently emerge within the first week suggests that not only phlebotomy-related blood loss but also coexisting mechanisms such as inflammation, suppressed erythropoiesis, and shortened erythrocyte lifespan contribute to the development of anemia [16]. The inflammatory response weakens erythropoiesis through multiple mechanisms [17]. Pro-inflammatory cytokines directly suppress erythroid proliferation in the bone marrow and simultaneously cause functional iron deficiency by increasing hepcidin expression via the interleukin-6-mediated pathway, leading to the limitation of iron release into the circulation [18]. Consequently, erythropoietic capacity is markedly reduced, impairing the maintenance of Hb levels under physiological stress. In our study, HAA developed in patients having significantly higher levels of inflammatory markers (CRP and ferritin), which demonstrated the role of inflammation in the generation of HAA. While ferritin retained its impact on the development of HAA in multivariate analysis including ferritin, CRP, and admission Hb levels; ICU length of stay; and DP volume, CRP lost its influence. So ferritin, as an acute phase reactant, might be involved in the evaluation of the inflammatory state of ICU patients alongside its role in iron status. 

In ICU patients, nutritional deficiencies such as vitamin B12, folate, and iron deficiencies also suppress erythropoiesis, resulting in the development of anemia [19]. Folate is an essential cofactor for DNA synthesis and erythropoiesis, particularly in tissues with high cellular turnover such as the bone marrow. Therefore, reduced folate levels during critical illness may further impair erythropoiesis, which is already suppressed by inflammation. In our study, although folate levels were found to be similar in patients developing HAA and those who do not, significantly higher folate levels in the group without Hb decline suggest that adequate folate status may support the preservation of the erythropoietic response. Indeed, factors such as insufficient enteral intake, impaired gastrointestinal absorption, and increased metabolic demands in ICU patients can lead to the rapid depletion of folate stores. Thus, declining folate levels should not be ignored in the intensive care setting to prevent the exacerbation of the development of HAA.

This study has several limitations. Due to its retrospective design, the frequency and timing of laboratory testing could not be standardized. Although all identifiable sources of bleeding were excluded based on clinical data, silent or microscopic blood losses could not be completely ruled out. Additionally, the volume of DP was calculated according to institutional nominal tube capacities, which may not fully reflect the actual fill levels and the true amount of blood drawn from patients. Furthermore, standardized severity-of-illness scores, such as APACHE II and SOFA, were not available for a sufficient proportion of patients. Therefore, residual confounding related to baseline illness severity cannot be completely excluded. Direct measures of erythropoiesis, including serum erythropoietin levels, reticulocyte responses, and bone marrow activity, were not available for the evaluation. Also there was a lack of data regarding the iron or vitamin supplements given to patients. Nevertheless, the large sample size, multivariable analytical approach, and comprehensive data structure focusing on etiological mechanisms provide strong evidence identifying significant and potentially modifiable factors contributing to the development of HAA in ICU patients.

In conclusion, HAA is a common and clinically significant problem in ICU patients, developing in approximately one-third of patients in our cohort. Our findings indicate that HAA is multifactorial, with lower admission Hb levels, longer ICU stays, higher ferritin levels, and greater total DP volume independently associated with its development. Higher ferritin and CRP levels, reflecting increased inflammatory burden, were observed in patients with HAA and in those with hemoglobin decline, supporting an important role of inflammation in the development of anemia. In addition, the independent association between greater total DP volume and HAA supports the contribution of iatrogenic blood loss to the development of HAA. Lower folate levels were also associated with hemoglobin decline and may further impair erythropoietic capacity. Overall, our findings suggest that patients with lower admission Hb levels and longer ICU stays are particularly susceptible to HAA, while inflammatory burden and diagnostic phlebotomy may also contribute to its development. More comprehensive prospective studies incorporating underlying causes of hospitalization, treatments, disease severity, and other potential confounding factors are warranted to better define the relative contributions of these factors to HAA.

## Figures and Tables

**Table 1:** Baseline and discharge characteristics of the study cohort.

Variable	Value
Total number of patients, *n*	321
Baseline characteristics	
➢ Age, years, median (range)	72 (18–102)
➢ Sex, *n* (%)	
▪ Female	145 (45.2%)
▪ Male	176 (54.8%)
➢ Admission Hb (g/dL), median (range)	14 (12–19.2)
➢ Admission eGFR (mL/min/1.73 m^2^), median (range)	82 (30–217)
➢ ICU length of stay, days, median (range)	6 (3–29)
➢ WBC (×10^3^/µL), median (range)	8.46 (1.81–23.2)
➢ PLT (×10^3^/µL), median (range)	228 (35–689)
➢ Ferritin (ng/mL), median (range)	88 (4–1500)
➢ CRP (mg/L), median (range)	29 (0.47–450)
➢ Vitamin B12 (pg/mL), median (range)	231 (50–1586)
▪ Normal, *n* (%)	271 (84.4%)
▪ Low, *n* (%)	35 (10.9%)
▪ Unknown, *n* (%)	15 (4.7%)
➢ Folate (ng/mL), median (range)	7.79 (1.98–25)
▪ Normal, *n* (%)	304 (94.7%)
▪ Low, *n* (%)	6 (1.9%)
▪ Unknown, *n* (%)	11 (3.4%)
Discharge characteristics	
➢ Discharge Hb (g/dL), median (range)	13 (8–17.6)
➢ Decrease in Hb (g/dL), median (range)	1 (0.1–8.8)
➢ Hospital-acquired anemia at discharge, *n* (%)	115 (35.8%)
➢ Total DP volume (mL), median (range)	58.2 (17.4–554.8)
➢ RBC transfusion, *n* (%)	3 (0.9%)

Data are presented as the median (range) or *n* (%). Hb: hemoglobin; CRP: C-reactive protein; DP: diagnostic phlebotomy; ICU: intensive care unit; eGFR: estimated glomerular filtration rate.

**Table 2:** Comparison of baseline characteristics between patients with and without hospital-acquired anemia.

Variable	HAA (*n* = 115)	No HAA (*n* = 206)	*p*-Value
➢ Age, years, median (range)	73 (23–98)	72 (18–102)	0.802
➢ Sex, *n* (%)			0.413
▪ Female	48 (41.7%)	97 (47.1%)	
▪ Male	67 (58.3%)	109 (52.9%)	
➢ ICU length of stay, days, median (range)	7 (3–29)	5 (3–22)	0.000
➢ Admission eGFR (mL/min/1.73 m^2^), median (range)	81 (31–217)	83 (30–179)	0.316
➢ Ferritin (ng/mL), median (range)	131 (8–1500	70 (4–1500)	0.000
➢ CRP (mg/L), median (range)	54 (0.6–441)	22 (0.4–450)	0.001
➢ Vitamin B12 (pg/mL), median (range)	251 (50–1586)	222 (63–1586)	0.176
➢ Vitamin B12, *n* (%)			
▪ Normal	94 (87.9%)	177 (89%)	
▪ Low	13 (12,1%)	22 (11%)	0.851
➢ Folate (ng/mL), median (range)	7.5 (1.98–25)	7.9 (2–24)	0.180
➢ Folate, *n* (%)			
▪ Normal	107 (98.2%)	197 (98%)	1.000
▪ Low	2 (1.8%)	4 (2%)	
➢ Admission Hb (g/dL), median (range)	13.6 (12–18.3)	14.5 (12–19.2)	0.000
➢ Total DP volume (mL), median (range)	70.6 (32.8–554.8)	51.8 (17.4–244.7)	0.000

Data are presented as the median (range) or a number (percentage). HAA: hospital-acquired anemia; Hb: hemoglobin; CRP: C-reactive protein; DP: diagnostic phlebotomy; ICU: intensive care unit; eGFR: estimated glomerular filtration rate.

**Table 3:** Univariate logistic regression analysis examining predictors of the development of HAA.

Variable	B	OR	95% CI	*p*-Value
ICU length of stay	0.158	1.172	1.102–1.246	0.000
Ferritin (ng/mL)	0.002	1.002	1.001–1.003	0.000
CRP (mg/L)	0.003	1.004	1.001–1.006	0.005
Admission Hb (g/dL)	−0.680	0.507	0.404–0.636	0.000
Total DP volume (mL)	0.019	1.019	1.011–1.027	0.000

OR: odds ratio; CI: confidence interval; CRP: C-reactive protein; DP: diagnostic phlebotomy.

**Table 4:** Multivariate logistic regression analysis examining predictors of the development of HAA.

Variable	B	OR	95% CI	*p*-Value
ICU length of stay	0.101	1.106	1.006–1.217	0.038
Ferritin (ng/mL)	0.002	1.002	1.001–1.003	0.01
CRP (mg/L)	0.002	1.002	0.999–1.005	0.214
Admission Hb (g/dL)	−0.874	0.417	0.316–0.550	0.000
Total DP volume (mL)	0.014	1.014	1.003–1.025	0.014

OR: odds ratio; CI: confidence interval; CRP: C-reactive protein; DP: diagnostic phlebotomy.

**Table 5:** Comparison of baseline characteristics between patients with and without hemoglobin decline.

Variable	Hb Decline (*n* = 258)	No Hb Decline (*n* = 63)	*p*-Value
➢ Age, years, median (range)	72.5 (18–102)	71 (48–98)	0.636
➢ Sex, *n* (%)			0.207
▪ Female	112 (43.4%)	33 (52.4%)	
▪ Male	146 (56.6%)	30 (47.6%)	
➢ ICU length of stay, days, median (range)	6 (3–29)	6 (3–14)	0.203
➢ Admission eGFR (mL/min/1.73 m^2^), median (range)	82 (30–217)	83 (33–119)	0.817
➢ Ferritin (ng/mL), median (range)	98.5 (4–1500)	60.5 (4–1500)	0.008
➢ CRP (mg/L), median (range)	40 (0.47–450)	18 (0.89–274)	0.01
➢ Vitamin B12 (pg/mL), median (range)	233 (50–1586)	220 (76–1586)	0.492
➢ Vitamin B12, *n* (%)			0.502
▪ Normal	214 (87.7%)	57 (91.94%)	
▪ Low	30 (12.3%)	5 (8.06%)	
➢ Folate (ng/mL), median (range)	7.25 (1.98–25)	9.25 (4–24)	0.001
➢ Folate, *n* (%)			0.604
▪ Normal	242 (97.6%)	62 (98.4%)	
▪ Low	6 (2.4%)	1 (1.6%)	
➢ Admission Hb (g/dL), median (range)	14 (12–19.2)	13.9 (12–17.4)	0.187
➢ Total DP volume (mL), median (range)	59.2 (17.4–554.8)	56.1 (19.4–165)	0.06

Data are presented as the median (range) or a number (percentage). Hb: hemoglobin; CRP: C-reactive protein; DP: diagnostic phlebotomy; ICU: intensive care unit; eGFR: estimated glomerular filtration rate.

**Table 6:** Univariate logistic regression analysis examining predictors of hemoglobin decline.

Variable	B	OR	95% CI	*p*-Value
Ferritin (ng/mL)	0.001	1.001	0.999–1.002	0.387
CRP (mg/L)	0.006	1.006	1.001–1.010	0.008
Total DP volume (mL)	0.011	1.011	1.001–1.022	0.027

OR: odds ratio; CI: confidence interval; CRP: C-reactive protein; DP: diagnostic phlebotomy.

**Table 7:** Multivariate logistic regression analysis examining predictors of hemoglobin decline.

Variable	B	OR	95% CI	*p*-Value
Total DP volume (mL)	0.009	1.009	0.999–1.020	0.068
CRP (mg/L)	0.005	1.005	1.001–1.009	0.023

**Note**: OR: odds ratio; DP: diagnostic phlebotomy; CI: confidence interval; CRP: C-reactive protein.

## Data Availability

The datasets used or analyzed in the current study are available from the corresponding author upon reasonable request.
